# Correlation of Anti-TULP1 Autoantibodies with Breast Cancer and Autoimmune Retinopathy

**DOI:** 10.3390/ijms26062569

**Published:** 2025-03-13

**Authors:** Collin Kaster, Sufang Yang, Grazyna Adamus

**Affiliations:** Ocular Immunology Laboratory, Casey Eye Institute, Oregon Health and Science University, Portland, OR 97239, USA; kasterc@ohsu.edu (C.K.); yangs@ohsu.edu (S.Y.)

**Keywords:** autoantibodies, autoimmune retinopathy, TULP1, breast cancer, epitope mapping

## Abstract

Autoantibodies have been implicated in the pathogenesis of autoimmune diseases, including autoimmune retinopathies. Our study aimed to identify retinal autoantigens recognized by serum autoantibodies (AAbs) in patients with visual disturbance. We evaluated 2453 serum samples for anti-retinal AAbs from patients with or without cancer and complaints of visual loss. Anti-TULP1 AAbs were more prevalent in the subset of women with breast cancer and vision loss. Epitope mapping was determined by ELISA using peptides covering the conservative carboxy terminal of TULP1, revealing major lineal epitopes within the sequences 334–341 and 480–488. We found no significant difference in the main epitope recognition between sera from patients with or without breast cancer. Although we show a correlation of anti-TULP1 AAbs with breast cancer, we found no TULP1 protein expression in breast cells, making this association unclear. In the retina, anti-TULP1 AAbs can disrupt the transport of proteins to outer segments and be involved in the degeneration of photoreceptors in a similar fashion to the degeneration induced by *TULP1* gene mutation. Nevertheless, the strong association of detectable anti-TULP1 AAbs in breast cancer patients with vision problems indicates its potential as a biomarker for cancer-associated autoimmune retinopathy.

## 1. Introduction

Autoimmune retinopathy (AIR) is an uncommon degenerative disease of the retina, which is associated with serum autoantibodies (AAbs) against retinal proteins. AAbs against different retinal proteins have been detected in the sera of people with vision loss, often leading to retinal degeneration. The most frequent anti-retinal AAbs were characterized in a recent study of cancer-associated retinopathy (CAR) [1]. In the current study, we showed that anti-TULP1 autoantibodies were more prevalent among women with breast cancer and vision loss than in other patients.

Tubby-like protein 1 (TULP1) is a somewhat relatively unknown antigen in autoimmune retinopathies. There are only two published articles describing the presence of anti-TULP1 AAbs in CAR. In 2000, Kikuchi et al. reported on anti-TULP1 AAbs in the serum of a patient with endometrial cancer and loss of vision [2]. They found that this patient also had anti-recoverin in addition to anti-TULP1 AAbs and that both were present prior to the diagnosis of endometrial cancer. The authors suggested that such AAbs blocked the function of the TULP1 protein in the photoreceptor cell in a manner similar to that of the mutated *TULP1* gene observed in retinitis pigmentosa (RP) [2]. In 2022, Chauhan et al. reported on a patient with CAR who also had AAbs against TULP1 that developed soon after the patient’s diagnosis of small-cell lung cancer [3]. The visual loss in their patient began following the initiation of atezolizumab therapy for cancer that, in effect, worsened the retinal dysfunction in this patient.

TULP1 belongs to the tubby family of proteins expressed in the retina and brain [4]. Pathogenic variants of the *TULP1* gene discovered in inhered retinal diseases (IRD) exhibit a broad range of severity and clinical presentations. Mutations in the *TULP1* gene have been shown to cause early-onset retinitis pigmentosa type 14 (RP14) and Leber congenital amaurosis (LCA15), associated with rapid progression and severe vision impairment [5,6]. In mouse models, genetic ablation of *TULP1* resulted in retinal degeneration, with marked deterioration of the photoreceptor layer, and, in effect, apoptosis of both rods and cones [4]. Over 40 different *TULP1* mutations have been reported in the Human Gene Mutation Database (RetNet—Retinal Information Network (2019) at: https://sph.uth.edu/retnet/, accessed on 24 September 2024), with most causing missense mutations in the conserved C-terminal tubby domain. Regardless of the type of mutation, clinical presentation is similar, including loss of peripheral vision, nyctalopia, and finally photoreceptor degeneration and blindness. Moreover, CAR patients with anti-TULP1 AAbs had similar presentation, including night blindness, and severe visual acuity loss, involving both rod and cone photoreceptors [7].

Anti-TULP1 AAbs were not investigated in RP patients with *TULP1* mutation, since such AAbs are a relatively new discovery. In this retrospective study, we analyzed the results of 2453 patients from a repository who were examined for AAbs against CAR panels collected between 2019 and 2022. We discovered that a considerable number of women with breast cancer and vision disturbance had serum anti-TULP1 AAbs, often coexisting with other anti-retinal AAbs, including a case of anti-recoverin and anti-Rab6A being present in the same patient.

In autoimmunity, autoantibodies might be generated early before, disease process has become symptomatic. These AAbs could be generated when TULP1 is released from damaged photoreceptor cells, leading to the production of AAbs, and then those AAbs could be implicated in secondary retinal degeneration. Because not much is known about the prevalence of anti-TULP1 AAbs in AIR and CAR as well in general healthy population, we focused this research on females with breast cancer and anti-TULP1 AAbs. We determined the antibody target cells in the retina as well as the major epitopes that were recognized by the patient sera. Our studies show the complexity of antibody-mediated autoimmunity in developing the possible pathogenic mechanism responsible for retinal degeneration in TULP1 autoimmunity.

## 2. Results

### 2.1. Occurrence of Anti-TULP1 AAbs in Breast Cancer

This retrospective study aimed to determine the prevalence of a novel autoantibody in autoimmune retinopathy. Most patients assessed had multiple specific AAbs occurring in the same patient. Out of 2453 individuals (average age 59), 607 (25%) had a history of different kinds of cancers. We evaluate the presence of AAbs against novel antigen TULP1 AAbs. Table 1 shows the presence of such AAbs in more frequent cancers in the population studied.

A total of 90 patients had a history of breast cancer, and anti-TULP1 AAbs were tested positive in 40% of this cohort. Seropositivity in breast cancer cases was significantly higher than in other cancers (*p* = 0.015). Active breast cancer was present in 18 cases, but in the majority of cases, cancer had been diagnosed years ago, prior to submitting serum for anti-retinal AAbs testing. In addition, some women had histories of additional malignancies, including gynecological, colon, bladder, renal, thyroid, melanoma, bone, and brain cancers (*n* = 20). Anti-recoverin and anti-Rab6 AAbs were detected along with anti-TULP1 AAbs, including 12% seropositivity for anti-recoverin and 13% for anti-Rab6 AAbs. Within 24 healthy subjects without vision problems, 3 were TULP1-positive.

Subsequently, our study focused on breast cancer patients. Over 30% of the patients had decreased visual acuity and field defects (36%). At baseline AAbs assessment, best corrected visual acuity varied from 20/30 to CF@6f. Patients mostly reported bilateral subjective vision loss, but 17 individuals reported unilateral loss. Table 2 summarizes patients’ main complaints based on the provided clinical notes, such as vision loss observed with a sudden onset in some individuals, as well as blurry vision, light sensitivity, constriction of visual fields (scotomas), and problems seeing in the dark (nyctalopia). Some patients had trouble recognizing colors (12%), with color vision fluctuating from 12/14 to 0/11 on Ishihara plates. Based on the ERG studies, decreased visual function was associated with abnormal cone and rod responses (18%). Fundus examination showed vascular attenuation (9%), retinal pigmentary changes (17%), retinal atrophy (14%), and optic nerve pallor (14%).

### 2.2. Anti-TULP1 AAbs Label Photoreceptor Inner Segments

**Figure 1:**
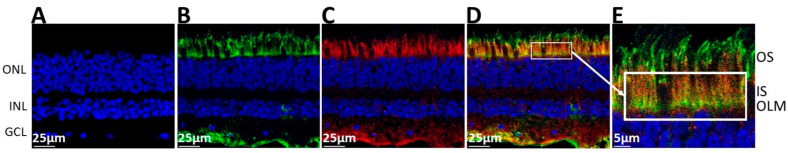
Representative immunofluorescent labeling images of human retina with human serum containing anti-TULP1 AAbs. (**A**) No antibody, DAPI-stained nuclei (blue), (**B**) staining with serum positive for anti-TULP1 antibody (green), (**C**) control staining with anti-TULP1 MAb (red), (**D**,**E**) double stain with anti-TULP1 MAb and TULP1-positive human sera showing overlapping binding (yellow); arrow indicates a larger magnification of a staining overlap between the inner segments of photoreceptor cells. Abbreviations: OS, outer segments; IS, inner segments; OLM, outer limiting membrane; ONL, outer nuclear layer; INL, inner nuclear layer; GCL, ganglion cell layer.

**Figure 2:**
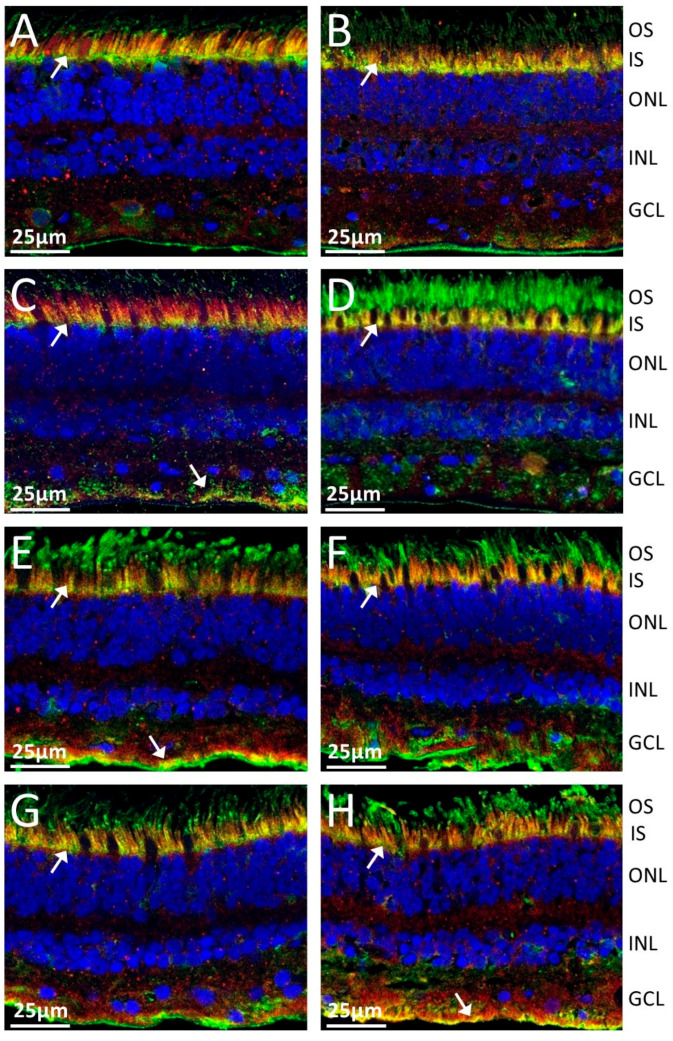
Representative images of immunofluorescent double labeling of outer retina with sera from presumed CAR and AR patients, specific for anti-TULP1 AAbs. (**A**,**B**)—AIR sera; (**C**–**H**)—CAR sera. Seropositive anti-TULP1 AAbs (green); commercial anti-TULP1 antibodies (red). Overlapping staining (yellow); DAPI stained nuclei (blue). Arrows point at double immunofluorescent staining of the inner segments in photoreceptor cells. Abbreviations: OS, outer segments; IS, inner segments; ONL, outer nuclear layer; INL, inner nuclear layer; GCL, ganglion cell layer.

The subcellular localization of TULP1 in the retina was performed in indirect immunofluorescence experiments. To elucidate whether anti-TULP1 sera would label human retinal tissue in the same fashion as commercial anti-TULP1 antibodies, we performed a double immunofluorescent staining. Serum anti-TULP1 AAbs labeled photoreceptor inner segments and outer limiting membrane, as well as the retinal ganglion and nerve fiber layer (Figure 1B; green). Serum anti-TULP1 antibodies immunolabeled the inner segments in the same fashion as specific anti-TULP1 antibodies (Figure 1C; red). Anti-TULP1 antibody (red color) colocalized with TULP1-specific serum (green color), shows overlapped staining of inner photoreceptor segments and the neuronal fiber layer in Figure 1D (yellow color). Figure 1E shows a magnification of the labeling of the inner segment (arrow) and outer limiting membrane. We tested eight randomly selected sera positive for TULP1 AAbs on the blot (Figure 2), including two from AIR patients without cancer (Figure 2A,B) and six from patients with different types of cancers (basal cell carcinoma, small cell lung cancer, breast cancer, renal cancer) (Figure 2C–H). Double immunofluorescent labeling shows that all anti-TULP1 sera stained the inner segment of the photoreceptor cells with some staining retinal ganglion cell and nerve fiber layers (yellow color). In summary, sera with anti-TULP1 antibodies labeled the same photoreceptor structures as commercial antibodies specific for TULP1 independently of whether they were from cancer patients or patients who had not been diagnosed with cancer.

### 2.3. Epitope Mapping for Anti-TULP1 AAbs

To examine lineal binding epitopes of human TULP1 protein we synthesized a set of peptides, 20 amino acids in length, overlapping the C-terminal TULP1 domain stretching from the amino acid 296 to 542 (Section 4.4). We randomly selected anti-TULP1 AAb containing sera from breast cancer patients (*n* = 14) and no cancer patients (*n* = 13) for epitope searching by ELISA. We found only three anti-TULP1 seropositive sera from normal subjects, but the titer was too low to produce meaningful data in ELISA epitope mapping experiments, and they are not included in the analysis.

**Figure 3:**
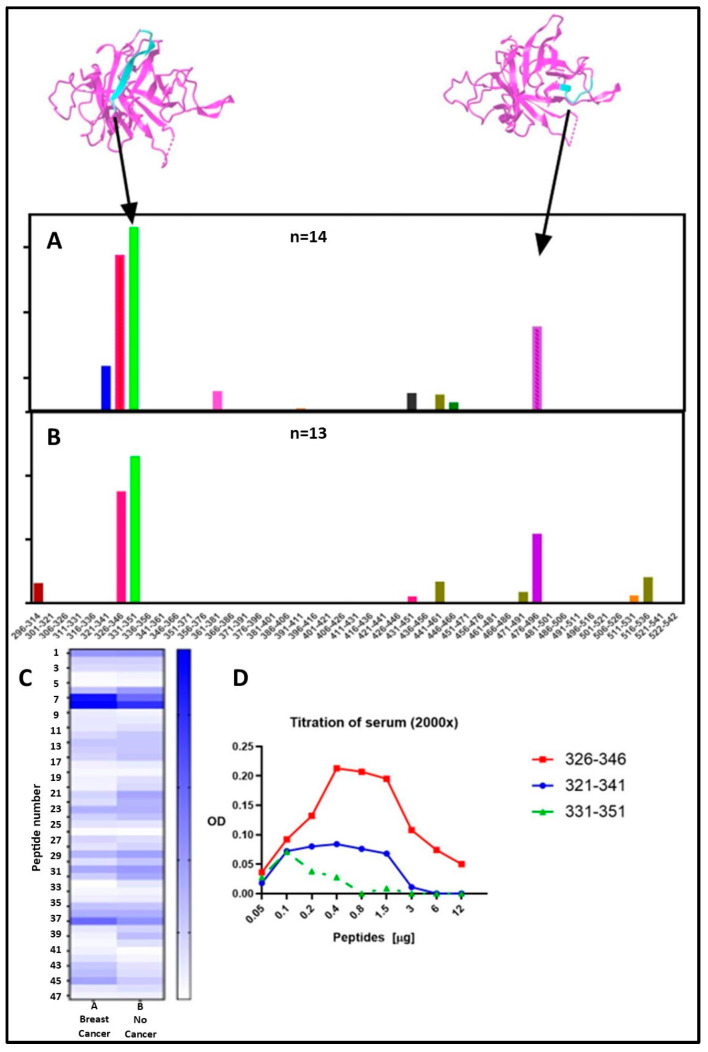
Comparison of epitope mapping of TULP1 carboxyl-terminal domain. (**A**,**B**)—bar graphs showing the summary of serum reaction against 20 mer peptides. Position of major epitopes shown in topology diagrams of the TULP1 COOH-terminal model (above the bars). Both major epitopes are exposed on the surface of TULP1 protein, with epitope 1 sequence 331–341 and epitope 2 sequence 480–487. (**C**) Heatmap analysis of breast CAR (*n* = 14) and AIR (*n* = 13) patient’s anti-TULP1 AAbs using GraphPad Prism software version 9. (**D**) Titration of serum antibodies against peptides within the TULP1 major epitope sequence 331–341. The peptide 321–341 was the most reactive with serum (red).

Results from the epitope mapping are shown in Figure 3. All serum AAbs recognized the same 2 major lineal epitopes in the TULP1 sequence independent of cancer diagnosis, and some minor epitopes specific to each group. The two groups evaluated were not statistically different (two-way ANOVA *p* = 0.3154). The sequence 331–338 (LYS-VAL-PHE-LEU-LEU-ALA-GLY-ARG) was the main Epitope 1 recognized, with 100% frequences for breast cancer samples and 85% for no-cancer samples. The second dominant Epitope 2 was recognized within the sequence 480–487 (GLY-LEU-VAL-THR-GLN-ALA-SER-VAL), with frequencies 93% and 92% for breast cancer and no-cancer samples, respectively. The calculated frequency is presented in Table 3.

Positions of major epitopes are shown in the topology diagrams of the TULP1 COOH-terminal model (above the bars), revealing that both immunogenic peptides are located on the surface of the protein with positively charged side chains, and therefore could, potentially, be accessible for antibody binding (Figure 3A). We analyzed the TULP1 predictive B cell epitopes using the IEDB Analysis Resource V2 Tool (IEDB, NIH, Bethesda, MD, USA). The major and minor epitopes from the experimental data overlapped with predicted linear determinants based on the protein C-terminal domain. Moreover, these AAbs targeted epitopes found to be close to the disease-causing mutants in RP, including Arg378His (human TULP1 numbering) and Ala496Thr [8]. These findings suggest that targeting the surface of this protein by AAbs in autoimmune retinopathies may induce similar processes, such as degeneration of photoreceptor cells, due to a gene mutation in RP.

To confirm the reactivity of serum containing anti-TULP1 an additional titration was performed using overlapping peptides sequences 321–341, 326–346, and 331–351 (Figure 3D). The peptides were twofold diluted from 0.05 µg to 12 µg and coated in ELISA plates, following by incubation with patient’s serum. An example for Epitope 1 is shown in Figure 3; anti-TULP1 serum reacted with all three peptides with the most reactive peptide 326–346, the sequence that was identified within the major epitope 331–338.

### 2.4. Concurrence of Anti-Rab6 and Anti-Recoverin Autoantibodies

**Figure 4:**
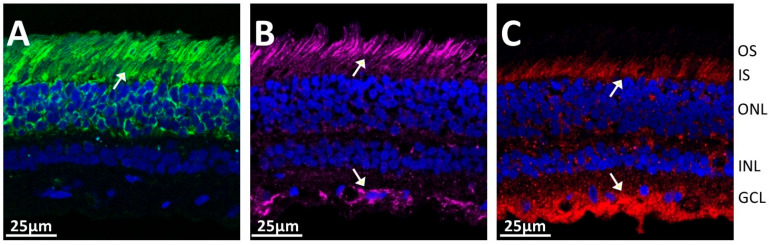
Comparison of immunofluorescent staining of human retina with (**A**) anti-Recoverin antibody (green), (**B**) anti-Rab6A MAb, (magenta), and (**C**) anti-TULP1 Mab (red), DAPI-nuclei staining (blue). Arrows point at retinal structures that are immunostained by specific antibodies as indicated in (**A**–**C**). Abbreviations: OS, outer segments; IS, inner segments; ONL, outer nuclear layer; INL, inner nuclear layer; GCL, ganglion cell layer.

In this cohort of TULP1 seropositive patients in women with breast cancer (*n* = 36/90), anti-recoverin (*n* = 12/90) and anti-Rab6 (*n* = 13/90) AAbs were found to be present along, with anti-TULP1, in the same patient. We sought to determine whether these proteins exist in the same retinal cells. Thus, we performed an immunofluorescent staining of human retina with the relevant commercial control antibodies against TULP1, Rab6, and recoverin. As is presented in Figure 4, each antibody shows a unique staining, but all labeled the outer and inner segments of photoreceptors cells. Anti-TULP1 antibody labeled the inner segments stronger, anti-recoverin labeled both outer and inner segments, and anti-Rab6 stained the outer segments in photoreceptor cells stronger. In addition, the ganglion cell layer was immunostained by anti-TULP1 and anti-Rab6 antibodies, indicating that these proteins could be released at the same time during retinal degeneration and stimulate immune responses.

**Figure 5:**
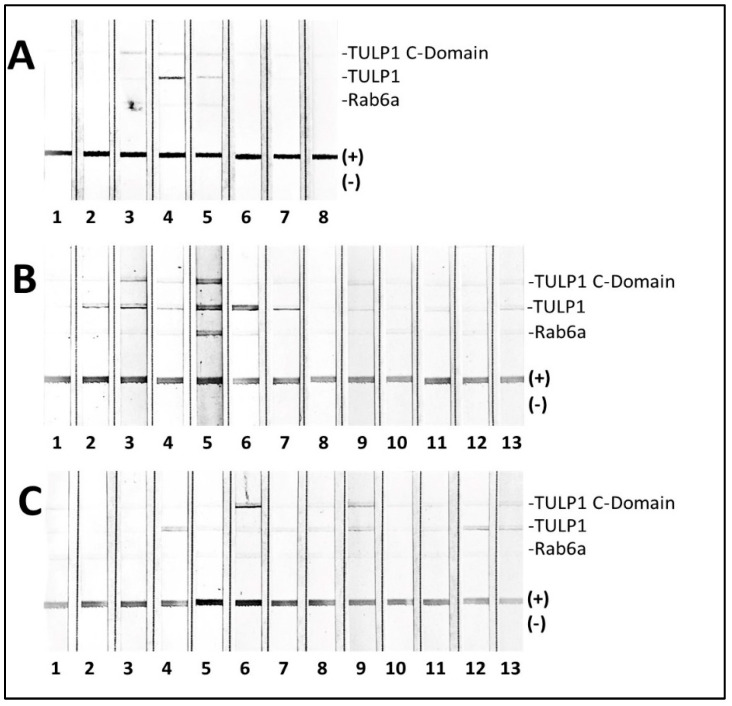
Immunoblotting of TULP1, C-domain TULP1, and Rab6 with serum antibodies of patients diagnosed with (**A**) lupus (*n* = 8), (**B**) retinitis pigmentosa (*n* = 13), and (**C**) uveitis (*n* = 13). The experiment was performed by printing those proteins directly on the membrane followed by blocking and incubation with serum AAbs as described in Methods. The positive control was protein retinal extract, and the negative control consisted of buffer.

In addition, we wanted to know whether the sera of patients diagnosed other ocular problems contain anti-TULP1 and anti-Rab6 AAbs. We analyzed three groups of patients diagnosed as follows: autoimmune uveitis (*n* = 13), retinitis pigmentosa (*n* = 13), and SLE (*n* = 8) groups. All RP subjects were collected before genetically testing was available. Immunoblots containing TULP1, its conservative C terminal domain, and Rab6 proteins were printed on the membrane. The results presented in Figure 5 show that the SLE group (A) had 2/8 seropositive TULP1 serum samples, with 6/13 in the RP group (B), including 2 subjects with AAbs against the conservative domain, and 4/13 in the uveitis group (C), with 2 against the carboxyl domain being anti-TULP1 positive. Rab6 was recognized only by one serum in the RP group (subject 5). This implies that the presence of anti-TULP1 AAbs are more distributed than was anticipated; however, their significantly high presence in breast cancer patients remains difficult to explain.

## 3. Discussion

Autoantibodies are part of the normal immune response, and sera from healthy subjects have large quantity of autoantibodies not linked to cancer or retina. These AAbs do not produce pathological effects for years. Moreover, women are highly predisposed to autoimmunity and generation of AAbs [9,10] and visual disturbance [11]. Furthermore, we and others have found AAbs in the sera of patients before cancer was diagnosed, suggesting that the breakdown of tolerance to tumor antigens occurs early in carcinogenesis [12,13].

Anti-TULP1 AAbs uniquely label the retina and may be generated in response to widespread retinal degenerations, including retinal atrophy, vessel attenuation, and RPE changes. It is possible that in our cohort of patients, AAbs against TULP1 developed in the pre-clinical stage in extremely low titers when patients were still asymptomatic; however, such AAbs might be detected in the later stages of their life when visual loss becomes evident [14]. The most interesting finding is that a significant number of women with diagnosed breast cancer were seropositive for anti-TULP1 AAbs. The presence of anti-retinal AAbs in women with current and past malignancy has been shown before, but the TULP1 antigen has not been studied until now [15]. It has been suggested that in presumed cancer-associated retinopathy, AAbs are likely made against tumor antigens like recoverin that cross-react with retina, and such antibodies might be responsible for eliminating tumors and at the same time targeting the retinal autoantigens, in effect causing photoreceptor degeneration [16,17,18,19].

In this study, we showed that our patient’s serum anti-TULP1 AAbs labeled the inner segments of retinal photoreceptors, which coincides with the previously published study of labeling this structure using the commercial control antibody anti-TULP1 antibody not serum AAbs [20,21]. In general, TULP1 protein was localized in the inner segments but also in retinal ganglions and Muller and RPE cells. In pathological conditions, this soluble protein may be released from damaged photoreceptors to help phagocytic removal of apoptotic cells or cellular debris either by RPE cells or recruited macrophages. The function of TULP1 in photoreceptors has not been fully explained, but this protein is likely functioning in protein trafficking in multiple photoreceptor compartments [22]. Both TULP1 and TUB are involved in the vesicular trafficking of several photoreceptor proteins from the inner segment to the outer segment [23]. TULP1-binding proteins might include cytoskeletal scaffold proteins, protein trafficking molecules, phototransduction cascade proteins, and GTPase-activating proteins [24]. Because the highest concentration of TULP1 is found in the inner segments, this suggests that TULP1 may be implicated in the transport of rod and cone opsins to outer segment proteins [25].

*TULP1* gene mutation also has a role in RP, which is characterized by retinal pigment deposits and the loss of rod photoreceptor cells followed by secondary loss of cone photoreceptors. It has been hypothesized that the mutated protein may not fold properly, which triggers photoreceptor death [26]. Such patients typically have night blindness and loss of peripheral visual field, which progresses to central vision loss at an early age. The first mutations in *TULP1* genes were discovered in inherited retinal diseases with the gene assigned to human chromosomal region 6p21·3, close to the RP14 locus responsible for an autosomal recessive form of retinitis pigmentosa [7]. Such mutations in the human *TULP1* gene cause vision loss in for 1–2% of RP cases in the world [26,27].

The pathogenic allele in the *TULP1* gene, with a single base pair substitution of lysine to arginine (p.K489R), was found to be adjacent to the major epitope for anti-TULP1 AAbs-binding. It is worth pointing out that symptoms in *TULP1* mutation RP are comparable with autoimmune retinopathies; however, some RP patients have early age of onset (~20 years old), rapid development, and reduced ERGs, while patients with presumed anti-TULP1 AIR or CAR are older and presented sudden loss of vision with rapid progression [28]. The average age of breast cancer patients in our study was 66 years old at presentation. The ERG findings in the clinical notes of our patients show a cone dysfunction consistent with previously reported cases of *tulp1*-related retinal dystrophy in mouse models.

The main question here is about the relationship of retinal TULP1 and breast cancer, or cancer in general. As of now, there is limited literature on the association of TULP proteins with cancer, although TULP1 is a possible ligand for the TAM family of kinases, and is overexpressed in cancers, including breast cancer [29]. Breast cancer is the most common cause of cancer-related mortality in women; however, the expression of TULP protein in breast carcinoma has not been reported. Our screening study of 37 malignant breast cancer tissue specimens for the presence of TULP1 was negative for the expression of this protein. One published study showed that expression levels of TULP3, a protein related to TULP1, were increased in colorectal cancer when compared to adjacent non-tumoral tissue [30]. The function of TULP3 was identified as a transcription factor and a primary regulator of carcinogenesis in pancreatic ductal adenocarcinoma [31]. Also, the higher a patient’s TULP3 expression, the poorer prognosis will be for PDAC patients [32].

Our results showed that AAbs against Rab6 and recoverin coincide with anti-TULP1 AAbs. We sought to understand the correlation between co-existence of anti-TULP1 AAbs with anti-Rab6a and anti-recoverin AAbs. All three proteins are located in photoreceptor cells and, when released during degenerative processes, can stimulate antibody production, including anti-recoverin, and anti-Rab6a that were found in CAR patients [33]. Rab6a and TULP1 are involved in protein dependent transport pathway in photoreceptor cells [25]. Rab6a is a low molecular weight protein member of the Ras superfamily of small GTPases, which may be involved in protein transport from the Golgi apparatus towards the endoplasmic reticulum (ER), and has low GTPase activity [34,35]. It has been suggested that the family members of the GTPase activating proteins collaborate with TULP1. In addition to in retina, Rab6a is found in the mammary epithelium, human brain, testis, prostate, and breast [35,36,37]. Aberrant expression Rab proteins have been demonstrated in various malignancies and implicated in pathogenesis [38]. Recoverin (23-kDa) is also a low molecular-weight protein, which is important in phototransduction as a calcium sensor predominantly found in photoreceptor outer segments and in bipolar cells and regulates rhodopsin phosphorylation through inhibition of rhodopsin kinase. Recoverin was found to also be expressed in cancer cells, which may be a critical step for triggering autoimmunity, leading to retinal degeneration [39]. In photoreceptors, TULP1 is colocalized with f-actin in the inner segments [40], where it may engage in intracellular trafficking of certain proteins, such as rhodopsin, and cone opsins between the inner and outer segments [25].

Our studies have limitations. First, our study is based on patients seen at tertiary centers, which can shift toward more complicated and severe AIR presentations compared with the general population. Furthermore, not all clinical tests were completed before submission for AAb testing for all samples. Second, the major strength of this study was the study size, but the population was diverse. Therefore, we focused on the occurrence of AAbs in 2453 patients whose sera were evaluated for anti-retinal Aabs; consequently, the sample of patients with breast cancer, vision disturbance, and anti-TULP1 is relatively small. Normally, healthy people had those antibodies, but at lower levels and prevalences. We believe that follow-ups will increase the likelihood of associations with relapses and antigen signatures being defined for those patients. Third, there is a clear association between the presence of anti-TULP1 AAbs and breast CAR; however, the role of TULP1 in breast carcinoma or other cancer tissues is still unknown. Also, a typical assumed limitation of epitope mapping by ELISA is the risk of missing conformational and discontinuous epitopes, which may not be represented by the linear peptide assay.

In summary, we studied biomarkers in patients with vision disturbance. We found that anti-TULP1 AAbs prevail in breast CAR; however, it is not clear how they contribute to the pathogenicity of retinopathy. However, blocking the function of this important retinal protein by specific AAbs can be as dramatic as a mutation in the *TULP1* gene [41]. The TULP1 Epitope 1, associated with specific phenotypes, could be considered a biomarker because of the high frequency of anti-TULP1 AAbs in breast CAR.

## 4. Materials and Methods

### 4.1. Serum Samples

These were retrospective studies on a cohort of 2453 patients that included 607 (25%) patients with various cancers. Serum samples that were collected between 2019 and 2022 and submitted to the Oregon Health and Science University Serum Repository with approval from the OHSU Institutional Review Board were evaluated. Samples were deidentified and stored at −20 °C, and results cannot be linked to subjects. In this cohort, a total of 283 samples were seropositive for the TULP1 protein (12%). Based on clinical notes obtained from the summary information submitted with specimens for antibody testing, we grouped the patients according to whether they had diagnosed cancer or were without diagnosed cancer. Samples lacking clinical information were excluded from this study. We determined that, in this cohort, the interval between the diagnosis of breast cancer and onset of vision disturbance was, on average, 5 years. For anti-TULP1 AAb controls, we tested 24 sera of healthy individuals without vision problems (average age 55) and patients diagnosed with systemic lupus erythematosus (SLE) (*n* = 8), retinitis pigmentosa (*n* = 13), and non-infectious uveitis (*n* = 13).

### 4.2. Immunoblotting

The sera were examined by a line immunoblot that contained a recombinant protein corresponding to the conservative domain of human TULP1, the residues 290 through 542 amino acids (NOVUS Biologicals, Centennial, CO, USA), and a panel of antigens that were frequently found in CAR [42]. The strips containing proteins were incubated for 1 h in 1:100 diluted serum. After washing, 1:2000 diluted anti-human IgG conjugated to alkaline phosphatase (Fisher Scientific, Waltham, MA, USA) was used for a 1 h incubation, and then a color reaction was developed with phosphatase substrate (Bio-Rad, Hercules, CA, USA). In addition, sera were re-tested using the entire sequence of TULP1 protein on a line blot (KACTUS, Waltham, MA, USA).

### 4.3. Double Fluorescent Immunolabeling

Donor eye tissue was obtained from the Eye Bank, and thin retinal sections (12 µm) were prepared by fixing using 4% paraformaldehyde for 5 min and then washing in phosphate-buffered saline (PBS). Next, the slides were boiled in 10 mM Citric Acid, with a pH of 6.0, for 15–30 min for antigen retrieval. After cooling and washing in PBS, sections were quenched in ethanol/acetic acid (2:1) at −20 °C for 20 min, followed by washing and incubation in 0.2% Triton X100 in PBS for 10 min. Then, the sections were blocked for 1 h in 10% normal goat serum, 1% bovine serum albumin, and 0.2% Tween 20 in PBS. Human sera, diluted at 1:50, were incubated overnight at 4 °C and then washed. Anti-human IgG conjugated to Alexa Fluor 488 (1:1000; Invitrogen, Waltham, MA, USA) was added for a 1 h incubation. For control, the tissue was incubated with the commercial control antibody rabbit anti-TULP1 antibodies (1:200, Origene, Rockville, MD, USA) [43] followed by incubation with anti-rabbit IgG conjugated to Alexa Fluor 594 (1:1000, Invitrogen). After a final wash, a 4′,6-diamidino-2-phenylindole (DAPI, Fluoromount-G, SouthernBiotech, Birmingham, AL, USA) with mounting reagent was added to seal the sections. The negative control was missing primary antibodies. Single staining for Rab6a and recoverin followed the same procedure and was incubated 1 h in rabbit anti-Rab6a antibodies (1:200, Novus, Chesterfield, MO, USA) or rat anti-recoverin antibodies (1:50, made by our lab) followed by incubation with anti-rabbit IgG conjugated to Alexa Fluor 594 (1:1000, Invitrogen) for 1 h. Images were acquired using a Leica TCS SP8 X confocal microscope (Leica, Wetzlar, Germany), with a Leica HC PL APO CS2 63×/1.40 and 40×/1.30 oil immersion objective (Leica), and Leica PMT and HyD hybrid detectors. Detection windows used were DAPI (410–504 nm), AF488 (504–593 nm), and Alexa Fluor 594 (603–751) nm. Brightness and contrast were adjusted using Leica LAS X software version 1.4.7.

### 4.4. Epitope Mapping by ELISA

We synthesized a library of 47 peptides, 20 mer in length, offset by 5 amino acids, covering the human carboxyl TULP1 domain from the residue 296 to 542 (Mimotopes Pty Ltd., Mulgrave, VIC, Australia) (Table 4). TULP1 peptides at 1 µg/well in duplicate were distributed into PVC 96-well plates (Fisher, Waltham, MA, USA), which were prepared as follows: soluble peptides were dissolved in water, insoluble peptides were first dissolved in 80% DMSO, then all peptides were diluted in 30% DMSO prior to being added to wells. Plates were coated with peptides overnight, and then washed and blocked with 1% nonfat milk in 0.05% Tween 20 in PBS buffer. Human sera (1:500) were added in duplicate to each well for 1 h, followed by anti-human IgG conjugated to horseradish peroxidase (1:1000, Fisher). An enzymatic reaction was developed for 30 min, and plates were read using a Bio-Rad plate reader with a wavelength of 450 nm and a reference wavelength of 630 nm.

### 4.5. Screening Breast Cancer Tissue for TULP1

A frozen tissue array of 37 malignant breast cancer tissue samples and 3 normal breast tissues (Bio-Chain, Newark, CA, USA) protected in OCT was used to screen the expression of TULP1 protein in cancer tissues with anti-TULP1 antibody. The slide was washed and incubated with 0.2% Triton X100 in PBS for 5 min. Then, the tissue was blocked in 10% normal goat serum, 1% bovine serum albumin, and 0.5% Tween 20 in PBS. Then, commercial rabbit anti-TULP1 antibodies (1:200, Origene) were added for a 1 h incubation with the tissue. After washing, anti-rabbit IgG conjugated to Alexa Fluor 594 (1:1000, Invitrogen) was added for 1 h. The images were acquired using a Leica TCS SP8 X confocal microscope, as before.

### 4.6. Statistical Analysis

The data are expressed as mean ± SEM for each group. Differences among groups were statistically analyzed using a two-tailed Fisher exact test, using Prism 4.0. Statistical significance was set at *p* < 0.05.

## 5. Conclusions

This study shows, for the first time, that a high prevalence of anti-TULP1 AAbs can be found in patients with vision disturbance and breast cancer compared to other patients. The question of whether anti-cancer immunity facilitates the production of these Aabs, leading to retinal degeneration, or the AAbs existed long before cancer was recognized in those patients remains open. The epitope determination for anti-TULP1 AAbs conducted in our studies shows that anti-TULP1 AAbs may be generated due to photoreceptor degeneration initiated by other processes, rather than initiated by an immune response to cancer antigens. TULP1 has, to date, not been shown to be expressed in breast cells, which further supports this notion. Despite this, detectable anti-TULP1 AAbs may serve as a biomarker for cancer-autoimmune retinopathy in breast cancer and may also be useful for breast cancer diagnosis.

## Figures and Tables

**Table 1:** TULP1 seropositivity in cancer patients.

Cancer	Total Cases (*n* = 430)	TULP1 Positivity (*n* = 76)	Positivity Rate (%)	*p* Value
BCC	32	6	19%	0.715
Bladder	17	4	24%	0.422
Breast	90	36	40%	0.015
Colon	16	1	6%	0.638
Kidney	21	4	19%	0.689
Lung	59	11	19%	0.748
Melanoma	103	5	5%	0.173
Ovarian	11	2	18%	0.640
Prostate	36	5	14%	1.000
Lymphoma	45	2	4%	0.333

**Table 2:** Ocular findings in breast cancer patients presented in the initial retinal autoantibody testing.

Ocular Findings	Number of Patients (*n* = 90)	Frequency (%)
Uveitis	7	8
Central vision loss	8	9
Peripheral vision loss	12	13
Color vision loss	11	12
Decreased visual acuity	27	30
Visual field defect (scotomas)	32	36
ERG defects	16	18
Retinal pigmentary changes	15	17
Retinal atrophy	13	14
Attenuated vessels	8	9
Optic disc pallor	13	14
Asymmetric loss	17	19
Sudden loss	4	4
Gradual loss	4	4
Progressive course	17	19
Blurry vision	21	23
Photopsia/photophobia	11	12
Nyctalopia (dark vision)	11	12

**Table 3:** Frequency of recognition of the major TULP1 epitopes.

Peptide	Frequency Breast Cancer Sera (*n* = 14)	Frequency No-Cancer Sera (*n* = 13)
326–346	93%	92%
331–351 (epitope 1)	100% (*p* = 0.00061)	85% (*p* = 0.0112)
471–491	93%	62%
476–496 (epitope 2)	93% (*p* = 0.000915)	92% (0.00172)

**Table 4:** Sequences of TULP1 peptides synthesized for epitope mapping.

Peptide Number	Peptide Position	Sequence
1	296–314	RPAPQGRTVRCRLTRDKKGM
2	301–321	GRTVRCRLTRDKKGMDRGMY
3	306–326	CRLTRDKKGMDRGMYPSYFL
4	311–331	DKKGMDRGMYPSYFLHLDTE
5	316–336	DRGMYPSYFLHLDTEKKVFL
6	321–341	PSYFLHLDTEKKVFLLAGRK
7	326–346	HLDTEKKVFLLAGRKRKRSK
8	331–351	KKVFLLAGRKRKRSKTANYL
9	336–356	LAGRKRKRSKTANYLISIDP
10	341–361	RKRSKTANYLISIDPTNLSR
11	346–366	TANYLISIDPTNLSRGGENF
12	351–371	ISIDPTNLSRGGENFIGKLR
13	356–376	TNLSRGGENFIGKLRSNLLG
14	361–381	GGENFIGKLRSNLLGNRFTV
15	366–386	IGKLRSNLLGNRFTVFDNGQ
16	371–391	SNLLGNRFTVFDNGQNPQRG
17	376–396	NRFTVFDNGQNPQRGYSTNV
18	381–401	FDNGQNPQRGYSTNVASLRQ
19	386–406	NPQRGYSTNVASLRQELAAV
20	391–411	YSTNVASLRQELAAVIYETN
21	396–416	ASLRQELAAVIYETNVLGFR
22	401–421	ELAAVIYETNVLGFRGPRRM
23	406–426	IYETNVLGFRGPRRMTVIIP
24	411–431	VLGFRGPRRMTVIIPGMSAE
25	416–436	GPRRMTVIIPGMSAENERVP
26	421–441	TVIIPGMSAENERVPIRPRN
27	426–446	GMSAENERVPIRPRNASDGL
28	431–451	NERVPIRPRNASDGLLVRWQ
29	436–456	IRPRNASDGLLVRWQNKTLE
30	441–461	ASDGLLVRWQNKTLESLIEL
31	446–466	LVRWQNKTLESLIELHNKPP
32	451–471	NKTLESLIELHNKPPVWNDD
33	456–476	SLIELHNKPPVWNDDSGSYT
34	461–481	HNKPPVWNDDSGSYTLNFQG
35	466–486	VWNDDSGSYTLNFQGRVTQA
36	471–491	SGSYTLNFQGRVTQASVKNF
37	476–496	LNFQGRVTQASVKNFQIVHA
38	481–501	RVTQASVKNFQIVHADDPDY
39	486–506	SVKNFQIVHADDPDYIVLQF
40	491–511	QIVHADDPDYIVLQFGRVAE
41	496–516	DDPDYIVLQFGRVAEDAFTL
42	501–521	IVLQFGRVAEDAFTLDYRYP
43	506–526	GRVAEDAFTLDYRYPLCALQ
44	511–531	DAFTLDYRYPLCALQAFAIA
45	516–536	DYRYPLCALQAFAIALSSFD
46	521–541	LCALQAFAIALSSFDGKLAC
47	522–542	CALQAFAIALSSFDGKLACE

## Data Availability

The original contributions presented in this study are included in the article. Further inquiries can be directed to the corresponding author.

## References

[1] Adamus G., Champaigne R., Yang S. (2020). Occurrence of major anti-retinal autoantibodies associated with paraneoplastic autoimmune retinopathy. Clin. Immunol..

[2] Kikuchi T., Arai J., Shibuki H., Kawashima H., Yoshimura N. (2000). Tubby-like protein 1 as an autoantigen in cancer-associated retinopathy. J. Neuroimmunol..

[3] Chauhan M.Z., Mansour H.A., Zafar M.K., Uwaydat S.H. (2022). Anti-Programmed Death Ligand-1 Induced Acute Vision Loss in a Patient With Cancer-Associated Retinopathy. Cureus.

[4] Carroll K., Gomez C., Shapiro L. (2004). Tubby proteins: The plot thickens. Nat. Rev. Mol. Cell Biol..

[5] den Hollander A.I., Roepman R., Koenekoop R.K., Cremers F.P. (2008). Leber congenital amaurosis: Genes, proteins and disease mechanisms. Prog. Retin. Eye Res..

[6] Majander A., Sankila E.M., Falck A., Vasara L.K., Seitsonen S., Kulmala M., Haavisto A.K., Avela K., Turunen J.A. (2023). Natural history and biomarkers of retinal dystrophy caused by the biallelic TULP1 variant c.148delG. Acta Ophthalmol..

[7] Wahl S., Magupalli V.G., Dembla M., Katiyar R., Schwarz K., Köblitz L., Alpadi K., Krause E., Rettig J., Sung C.-H. (2016). The Disease Protein Tulp1 Is Essential for Periactive Zone Endocytosis in Photoreceptor Ribbon Synapses. J. Neurosci..

[8] Gu S., Lennon A., Li Y., Lorenz B., Fossarello M., North M., Gal A., Wright A. (1998). Tubby-like protein-1 mutations in autosomal recessive retinitis pigmentosa. Lancet.

[9] Fairweather D., Rose N.R. (2004). Women and autoimmune diseases. Emerg. Infect. Dis..

[10] Klein S.L., Flanagan K.L. (2016). Sex differences in immune responses. Nat. Rev. Immunol..

[11] Zetterberg M. (2016). Age-related eye disease and gender. Maturitas.

[12] Adamus G., Yang S., Weleber R.G. (2016). Unique epitopes for carbonic anhydrase II autoantibodies related to autoimmune retinopathy and cancer-associated retinopathy. Exp. Eye Res..

[13] Fang H.-S., Yang C.-S., Cheng C.-K., Wang Y.-S. (2024). Cancer-associated retinopathy as an initial presentation of gynecologic small-cell carcinoma. Taiwan J. Ophthalmol..

[14] Deane K.D. (2014). Preclinical Rheumatoid Arthritis (Autoantibodies): An Updated Review. Curr. Rheumatol. Rep..

[15] Adamus G. (2015). Latest updates on antiretinal autoantibodies associated with vision loss and breast cancer. Investig. Ophthalmol. Vis. Sci..

[16] Lacombe J., Mangé A., Solassol J. (2014). Use of autoantibodies to detect the onset of breast cancer. J. Immunol. Res..

[17] Qiu J., Keyser B., Lin Z.T., Wu T. (2018). Autoantibodies as Potential Biomarkers in Breast Cancer. Biosensors.

[18] Bazhin A.V., Savchenko M.S., Shifrina O.N., Demoura S.A., Chikina S.Y., Jaques G., Kogan E.A., Chuchalin A.G., Philippov P.P. (2004). Recoverin as a paraneoplastic antigen in lung cancer: The occurrence of anti-recoverin autoantibodies in sera and recoverin in tumors. Lung Cancer.

[19] Polans A.S., Adamus G. (1995). Recoverin is the tumor antigen in cancer-associated retinopathy. Behav. Brain Sci..

[20] Milam A.H., Hendrickson A.E., Xiao M., Smith J.E., Possin D.E., John S.K., Nishina P.M. (2000). Localization of tubby-like protein 1 in developing and adult human retinas. Investig. Ophthalmol. Vis. Sci..

[21] Hagstrom S.A., Duyao M., North M.A., Li T. (1999). Retinal degeneration in tulp1-/- mice: Vesicular accumulation in the interphotoreceptor matrix. Investig. Ophthalmol. Vis. Sci..

[22] Ebke L.A., Sinha S., Pauer G.J.T., Hagstrom S.A. (2021). Photoreceptor Compartment-Specific TULP1 Interactomes. Int. J. Mol. Sci..

[23] Remez L., Cohen B., Nevet M.J., Rizel L., Ben-Yosef T. (2020). TULP1 and TUB Are Required for Specific Localization of PRCD to Photoreceptor Outer Segments. Int. J. Mol. Sci..

[24] Ebke L.A., Pauer G.J.T., Willard B., Hagstrom S.A. (2016). A novel approach to identify photoreceptor compartment-specific tulp1 binding partners. Adv. Exp. Med. Biol..

[25] Grossman G.H., Watson R.F., Pauer G.J.T., Bollinger K., Hagstrom S.A. (2011). Immunocytochemical evidence of Tulp1-dependent outer segment protein transport pathways in photoreceptor cells. Exp. Eye Res..

[26] Bodenbender J.P., Marino V., Bethge L., Stingl K., Haack T.B., Biskup S., Kohl S., Kühlewein L., Dell’Orco D., Weisschuh N. (2023). Biallelic Variants in TULP1 Are Associated with Heterogeneous Phenotypes of Retinal Dystrophy. Int. J. Mol. Sci..

[27] Bonilha V.L., Rayborn M.E., Bell B.A., Marino M.J., Beight C.D., Pauer G.J., Traboulsi E.I., Hollyfield J.G., Hagstrom S.A. (2015). Retinal histopathology in eyes from patients with autosomal dominant retinitis pigmentosa caused by rhodopsin mutations. Graefes Arch. Clin. Exp. Ophthalmol..

[28] Paloma E., Hjelmqvist L., Bayés M., García–Sandoval B., Ayuso C., Balcells S., Gonzàlez–Duarte R. (2000). Novel Mutations in the TULP1 Gene Causing Autosomal Recessive Retinitis Pigmentosa. Investig. Ophthalmol. Vis. Sci..

[29] Wu X., Liu X., Koul S., Lee C.Y., Zhang Z., Halmos B. (2014). AXL kinase as a novel target for cancer therapy. Oncotarget.

[30] Sartor I.T.S., Recamonde-Mendoza M., Ashton-Prolla P. (2019). TULP3: A potential biomarker in colorectal cancer?. PLoS ONE.

[31] Fanous I., Dillon P. (2015). Paraneoplastic neurological complications of breast cancer. Exp. Hematol. Oncol..

[32] Sartor I.T., Zeidán-Chuliá F., Albanus R.D., Dalmolin R.J., Moreira J.C. (2014). Computational analyses reveal a prognostic impact of TULP3 as a transcriptional master regulator in pancreatic ductal adenocarcinoma. Mol. Biosyst..

[33] Yang S., Dizhoor A., Wilson D.J., Adamus G. (2016). GCAP1, Rab6, and HSP27: Novel Autoantibody Targets in Cancer-Associated Retinopathy and Autoimmune Retinopathy. Trans. Vis. Sci. Technol..

[34] Liu S., Storrie B. (2012). Are Rab Proteins the Link Between Golgi Organization and Membrane Trafficking?. Cell Mol. Life Sci..

[35] Cayre S., Faraldo M.M., Bardin S., Miserey-Lenkei S., Deugnier M.A., Goud B. (2020). RAB6 GTPase regulates mammary secretory function by controlling the activation of STAT5. Development.

[36] Young J., Menetrey J., Goud B. (2010). RAB6C is a retrogene that encodes a centrosomal protein involved in cell cycle progression. J. Mol. Biol..

[37] Erol Ö.D., Şenocak Ş., Aerts-Kaya F. (2024). The Role of Rab GTPases in the development of genetic and malignant diseases. Mol. Cell Biochem..

[38] Anand S., Khan M.A., Khushman M., Dasgupta S., Singh S., Singh A.P. (2020). Comprehensive Analysis of Expression, Clinicopathological Association and Potential Prognostic Significance of RABs in Pancreatic Cancer. Int. J. Mol. Sci..

[39] Adamus G. (2003). Autoantibody-induced apoptosis as a possible mechanism of autoimmune retinopathy. Autoimmun. Rev..

[40] Xi Q., Pauer G.J.T., Marmorstein A.D., Crabb J.W., Hagstrom S.A. (2005). Tubby-like Protein 1 (TULP1) Interacts with F-actin in Photoreceptor Cells. Investig. Ophthalmol. Vis. Sci..

[41] Xi Q., Pauer G.J., Ball S.L., Rayborn M., Hollyfield J.G., Peachey N.S., Crabb J.W., Hagstrom S.A. (2007). Interaction between the photoreceptor-specific tubby-like protein 1 and the neuronal-specific GTPase dynamin-1. Invest. Ophthalmol. Vis. Sci..

[42] Adamus G. (2020). Current techniques to accurately measure anti-retinal autoantibodies. Expert. Rev. Ophthalmol..

[43] Adamus G., Amundson D. (1996). Epitope recognition of recoverin in cancer associated retinopathy: Evidence for calcium-dependent conformational epitopes. J. Neurosci. Res..

